# Chemical Composition, Anti-Tyrosinase and Antioxidant Potential of Essential Oils from *Acorus calamus* (L.) and *Juniperus communis* (L.)

**DOI:** 10.3390/molecules30112417

**Published:** 2025-05-31

**Authors:** Hubert Sytykiewicz, Iwona Łukasik, Sylwia Goławska

**Affiliations:** Faculty of Natural Sciences, Institute of Biological Sciences, University of Siedlce, 14 Prusa St., 08-110 Siedlce, Poland; iwona.lukasik@uws.edu.pl (I.Ł.); sylwia.golawska@uws.edu.pl (S.G.)

**Keywords:** antioxidant capacity, anti-tyrosinase activity, essential oil, *Acorus calamus* rhizome, *Juniperus communis* cone-berry

## Abstract

The essential oils (EOs) represent a natural source of diverse phytoconstituents that may exert a wide range of health-promoting effects, including antioxidative, anti-inflammatory, antimicrobial, and immunomodulatory activities. Compounds with antioxidative and anti-tyrosinase properties present in EOs may suppress excessive melanin production and protect skin cells from oxidative stress factors that often aggravate the pigmentation process. *Acorus calamus* L. and *Juniperus communis* L. plants have been traditionally used in phytotherapy, either individually or in combination. However, the biological and pharmacological effects of the essential oils derived from *A. calamus* rhizomes (EOA) and *J. communis* cone-berries (EOJ) remain underexplored. This study aimed to evaluate (1) the chemical composition of both EOA and EOJ using the gas chromatography–mass spectrometry (GC-MS) technique; (2) the anti-tyrosinase activity of the two examined EOs; and (3) their antioxidant potential against DPPH and ABTS free radicals. In addition, the anti-tyrosinase and antioxidant activities of mixtures of EOA and EOJ were also investigated. GC-MS analyses identified 48 and 81 chemical compounds in the EOA and EOJ, respectively. The main constituents of the EOA were sesquiterpenoids, including acorenone (18.1%), preisocalamendiol (12.0%), shyobunone (7.5%), and isoshyobunone (5.7%). In contrast, EOJ was primarily composed of α-pinene (22%), a monoterpene. In vitro analyses demonstrated that both individual and combined EOs exhibited notable antioxidant and anti-tyrosinase activities. The health-promoting potential of these EOs is discussed.

## 1. Introduction

The essential oils (EOs) exhibiting antioxidant properties are increasingly used in the pharmaceutical and cosmetics industries to produce anti-aging and skincare formulations. These compounds may slow the skin aging process and protect against UV radiation and environmental pollutants. In addition to their antioxidant effects, EOs may also possess anti-tyrosinase, anti-inflammatory, and antimicrobial properties [1,2,3]. The identification and investigation of EOs exhibiting both antioxidant and anti-tyrosinase activities may represent an important step in the development of skincare products and effective natural therapies for treating hyperpigmentation [1].

*Acorus calamus* L. (Acoraceae family) is a perennial, aromatic, and polymorphic herb with a wide geographic distribution (Europe, Asia, India, and North America) [4]. Previous studies have demonstrated that EOs extracted from *A. calamus* exhibit diverse antibacterial, antifungal, and antiviral activities, as well as repellent and biocidal properties against arthropods. Additionally, these EOs have shown a range of antioxidant and anti-inflammatory activities [4,5,6]. *Juniperus communis* L. (Cupressaceae family) is a shrub or small tree (typically up to about 10 m high) with a native range that spans the Northern Hemisphere, including Europe, Asia, and North America [7]. In Europe, it is widely distributed and is a natural component of various ecosystems such as forests and heathlands. It is also cultivated in dry, sunny habitats with poor, sandy, and acidic soils [7]. Studies have shown that EOs or their individual constituents derived from *J. communis* display a broad spectrum of biological activities, including diuretic, antimicrobial, anti-inflammatory, antioxidant, and antidiarrheal effects [7,8,9].

Crushed or powdered rhizomes of *A. calamus* and cone-berries of *J. communis* have traditionally been used in phytotherapy in the form of various aqueous and hydro-alcoholic extracts (such as infusions, decoctions, and tinctures) [4,10,11]. Both plant materials contain a unique composition of chemical constituents (e.g., flavonoids, terpenoids, alkaloids, phenylpropanoids), and when used together, they may synergistically enhance each other’s efficacy, especially during topical applications in the form of rubs, poultices, bath additives, or cosmetics [4,5,6,12,13].

Tyrosinase (EC 1.14.18.1) is a copper-containing oxidoreductase involved in melanin biosynthesis, occurring in melanocytes within human skin [14,15]. The central role of tyrosinase in melanogenesis is associated with two sequential reactions catalyzed by this enzyme. The first reaction involves the oxidation of L-tyrosine to L-3,4-dihydroxyphenylalanine (L-DOPA), and the second is the formation of dopaquinone, which may subsequently be converted into melanin pigments [14]. Elevated tyrosinase activity has been associated with various skin hyperpigmentation disorders, such as melasma, post-inflammatory pigmentary alteration (PIPA), freckles, and solar lentigines [15]. Topical application of tyrosinase inhibitors is one of the key strategies for preventing or treating different forms of skin hyperpigmentation [16,17]. Essential oils isolated from plant materials usually contain a wide spectrum of chemical constituents, often unique in terms of their structure and physicochemical properties, which may interact with each other within a mixture in synergistic, additive, or antagonistic ways [15]. Therefore, there is a need to screen a broad range of EOs and subsequently identify the most active components with high anti-tyrosinase potential, which could be further subjected to studies aimed at evaluating their potential toxicity or allergenic effects.

Despite an increasing number of scientific reports on the health-promoting and/or therapeutic effects of aqueous or alcoholic extracts of *A. calamus* and *J. communis* [4,5,6,18], the properties of essential oils isolated from these plants have been studied to only a limited extent [13]. Geographical origin and complex environmental factors affecting plant growth and development have a significant impact on the chemical composition and potential bioactivity of EOs. Therefore, it is important to study EOs extracted from plants collected in different regions and to identify those samples exhibiting the highest biological activity [5,6,19].

To the best of our knowledge, there are no available reports regarding the anti-tyrosinase activity of the two examined essential oils derived from *A. calamus* rhizomes and *J. communis* cone-berries (i.e., EOA and EOJ, respectively), either individually or in combination. Moreover, no information is available concerning the antioxidant effects of mixtures composed of these two EOs. The aim of the present study was to investigate (1) the chemical composition of the examined EOs, determined using gas chromatography–mass spectrometry (GC-MS); (2) the anti-tyrosinase activity of the two EOs; and (3) their antioxidant potential against DPPH^•^ and ABTS^•+^ free radicals. The secondary objective was to evaluate both the anti-tyrosinase and antioxidant activities of mixtures of the two EOs.

## 2. Results and Discussion

### 2.1. Chemical Composition of the Essential Oils Extracted from A. calamus and J. communis Plants

The gas chromatography–mass spectrometry (GC-MS) analyses enabled the identification of 48 and 81 chemical constituents in the essential oils extracted from *Acorus calamus* L. rhizomes and *Juniperus communis* L. cone-berries (i.e., EOA and EOJ), respectively (Table 1 and Table 2, Figures S1 and S2).

The major components of EOA were sesquiterpenoids, including acorenone (18.1%), preisocalamendiol (12.0%), shyobunone (7.5%), and isoshyobunone (5.7%), while the phenylpropanoid β-asarone was present in a slightly lower amount (4.2%) (Table 1, Figure S1). In addition, α-selinene, β-gurjunene, and isocalamendiol (sesquiterpenoids), as well as camphor (a bicyclic monoterpene ketone), were detected in concentrations ranging from 3% to 4%. The remaining identified constituents were present at levels below 3%.

In the essential oil derived from *J. communis* cone-berries (EOJ), the predominant compound was α-pinene (22%), a monoterpene. Other compounds, including the sesquiterpenoid caryophyllene oxide, the monoterpenes p-cymene and β-myrcene, the monoterpenoid alcohol terpinen-4-ol, and the sesquiterpene alcohol spathulenol, were found in relatively lower but comparable amounts, ranging from 3.1% to 3.7% (Table 2, Figure S2).

Environmental factors affecting plant growth and development (e.g., pH, physico-chemical properties and microbiome of soil, water availability, light intensity, topography, altitude, interspecies interactions, weather conditions, and exogenous stressors) indirectly influence the qualitative and quantitative composition of essential oil constituents [7,9,20]. Therefore, before conducting a variety of in vitro tests to determine the bioactivity of EOs, it is crucial to identify the major chemical compounds and their relative proportions. However, it should be emphasized that the extraction method used may also influence the chemical composition of the EOs [21]. In this study, we employed the commonly used hydrodistillation method to extract the two essential oils from the tested plant materials [20,21]. GC-MS analyses revealed that the EOA and EOJ represented acorenone-rich and α-pinene-rich chemotypes, respectively. The acorenone-rich chemotype of EOA is characterized by acorenone as the dominant compound and is more frequently used in traditional medicine and aromatherapy compared to the asarone-rich chemotype of *A. calamus*, which is increasingly applied in pest management and other biocidal uses [22]. Recent studies published over the last decade have demonstrated significant differences in the chemical composition and relative proportions of compounds between the two main chemotypes of *A. calamus* described above [10,23,24]. It has been reported that the amount of asarone in the rhizomes of *A. calamus* is linked to its ploidy and genetic diversity [25]. Most of the EOs from rhizomes of triploid karyotypes of this plant species contain 3–19% β-asarone, while oils from triploid plants may contain up to 96% of this compound [25]. Süzgeç-Selçuk et al. [23] reported that samples of EOs extracted from several localities in Turkey represented the asarone-rich chemotype and differed significantly in the content of major chemical constituents (approx. 15–16% β-asarone, 7–16% acorenone B, about 7–14% camphor, 6–8% camphene, 3–7% epi-isoshyobunone, and 1.5–4.6% shyobunone). Additionally, Loying et al. [24] performed hydrodistillation of EOs from rhizomes of *A. calamus* populations in Northeast India. According to these researchers, GC-MS analyses identified β-asarone as the dominant constituent in the EOA, occurring in high amounts (about 82%), while other components were detected at considerably lower concentrations (e.g., calarene, euasarone, and 2-methoxy-3-allylphenol at 1.9–2.4%).

The compound β-asarone has been documented for its genotoxic and potentially carcinogenic properties [26], which has led to its prohibition in cosmetic formulations under the European Commission Regulation (EC) No. 1223/2009 [27]. In light of these regulatory and toxicological concerns, the study by Szczeblewski et al. [28] is of particular relevance, as it describes the development and optimization of a centrifugal partition chromatography (CPC) protocol aimed at the selective removal of β-asarone from essential oils derived from *A. calamus* rhizomes. This line of investigation highlights the potential of chromatographic refinement techniques to enhance the safety profile of essential oils by eliminating constituents with recognized toxicological risks, thereby broadening their possible applicability in pharmaceutical or cosmetic contexts. Importantly, Raal et al. [29] revealed that among the EOs isolated from *A. calamus* rhizomes grown in Estonia, one sample represented the β-asarone-rich chemotype (approx. 85% β-asarone, indicating a tetraploid cytotype), whereas two other EO samples contained acorenone as the major constituent (approx. 22–27%), indicating the triploid karyotype of the plants. These two samples also contained lower levels of β-asarone (approx. 9–10%), 8–14% shyobunone, about 8% preisocalamendiol, and up to 5% isoacorone.

Furthermore, Hrytsyna et al. [7] investigated the chemical composition of *J. communis* cone-berries collected from various localities in Albania, Bosnia, Bulgaria, the Czech Republic, Slovakia, and Slovenia. These authors reported significant differences in the levels of total phenolic compounds, organic acids, and sugars in extracts derived from the cone-berries, depending on geographic location and altitude. Essential oils extracted from the cone-berries of European populations of *J. communis* are predominantly characterized by the α-pinene chemotype [8]. Salamon et al. [9] further demonstrated that the major constituents of EOs extracted from *J. communis* cone-berries grown in Slovakia included α-pinene (38–61%), β-myrcene (8–11%), and sabinene (3.5–22%). In a separate study, Höferl et al. [8], using GC-MS and gas chromatography with flame ionization detection (GC-FID), found that the EO isolated from *J. communis* cone-berries in Bulgaria primarily contained α-pinene (approx. 51%), along with other monoterpenes such as myrcene, sabinene, and β-pinene (approx. 5–6%).

### 2.2. Assessment of Anti-Tyrosinase Potential of the Tested Essential Oils

**Figure 1:**
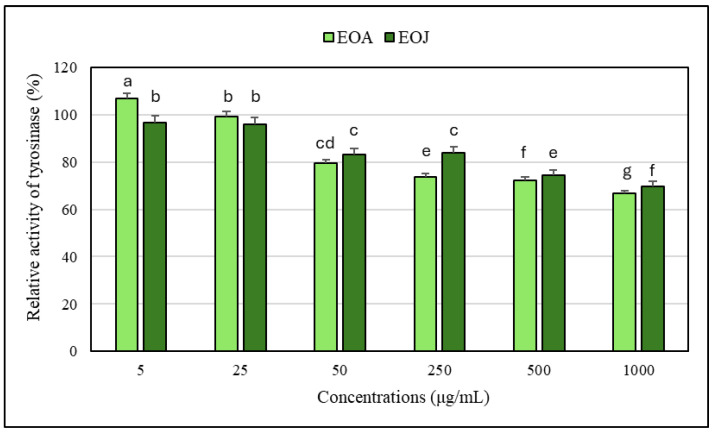
Effects of the tested concentrations of single essential oils on tyrosinase activity. EOA and EOJ—essential oils extracted from *Acorus calamus* L. rhizomes and *Juniperus communis* L. cone-berries, respectively. Tyrosinase activity level measured in the absence of the EOs (control) was considered 100% enzyme activity. Distinct letters above the error bars indicate significant differences in the means (±SD) of tyrosinase activity between the samples, as determined by Tukey’s test (*p* < 0.05).

**Figure 2:**
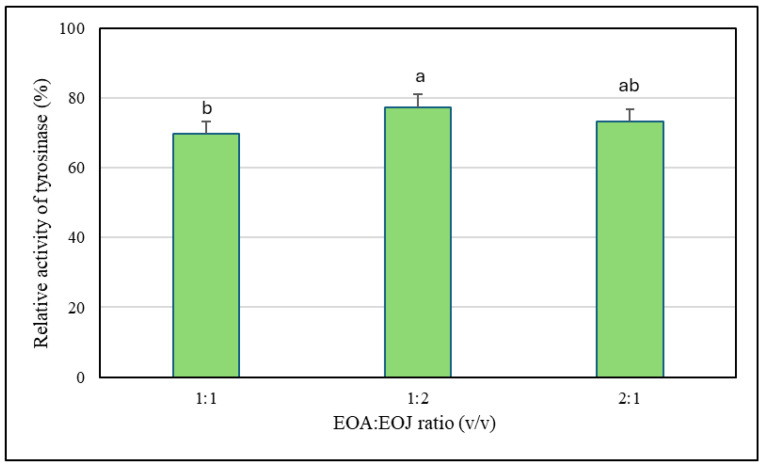
The impact of the essential oil mixtures (EOA and EOJ) on tyrosinase activity. EOA and EOJ—essential oils extracted from *Acorus calamus* L. rhizomes and *Juniperus communis* L. cone-berries, respectively. Tyrosinase activity level measured in the absence of the EOs (control) was considered 100% enzyme activity. The proportions 1:1, 1:2, and 2:1 refer to the volume ratios of EOA to EOJ (*v*/*v*) in the tested mixtures. Distinct letters above the error bars indicate significant differences in the means (±SD) of tyrosinase activity between the samples, as determined by Tukey’s test (*p* < 0.05).

It has been shown that the tested ethanolic solutions of EOA at 50, 250, 500, and 1000 µg/mL exhibited a 2–10% higher inhibitory effect against the tyrosinase enzyme compared to the same concentrations of the other essential oil examined (i.e., EOJ) (Figure 1). Moreover, these ethanolic solutions of both essential oils were characterized by dose-dependent anti-tyrosinase activity in relation to the enzyme control (without the addition of the EOs). The degree of inhibition of tyrosinase activity caused by the tested concentrations of EOs ranged from 1 to 33% for EOA and 3 to 30% for EOJ, respectively. However, it should be emphasized that the lowest tested concentration of EOA (i.e., 5 μg/mL) stimulated tyrosinase activity (approx. 7% increase compared to the enzyme control), whereas the EOJ at the same concentration showed approximately 3% inhibition of the enzyme activity. In addition, concentration of 25 µg/mL of the EOA did not affect the enzyme activity, whereas the EOJ caused about 4% inhibition compared to the control (Figure 1). Furthermore, kojic acid (140 µg/mL) was used as the inhibitor control (IC) in the anti-tyrosinase assay, leading to about 17% inhibition of the enzyme activity. Importantly, the anti-tyrosinase activity of mixtures of the two tested EOs in the following ratios of EOA and EOJ—1:1, 1:2, and 2:1 (*v*/*v*)—was also evaluated. The mixture of the tested oils (EOA:EOJ) in a 1:1 (*v*/*v*) ratio exhibited the highest degree of tyrosinase inhibition (approx. 30%), whereas a 2:1 (*v*/*v*) ratio showed a slightly lower inhibition (approx. 27%), and the lowest enzyme activity suppression (about 23%) was recorded for the 1:2 mixture (Figure 2). As summarized in Table 3, all tested volume ratios (1:1, 1:2, and 2:1 of EOA to EOJ) resulted in CI values below 1, indicating varying degrees of synergistic or additive effects. The 1:1 mixture (CI = 0.805) showed moderate synergism, while the 2:1 (CI = 0.904) and 1:2 (CI = 0.920) mixtures exhibited slight synergism or near-additive interactions. These results suggest that combining EOA and EOJ enhances the inhibition of tyrosinase activity more effectively than the individual essential oils alone, with the most balanced ratio (1:1) yielding the greatest synergistic effect.

This is the first report demonstrating the anti-tyrosinase activity of EOs isolated from *A. calamus* and *J. communis* (both as single EOs and their mixtures). However, studies conducted by Jegal et al. [31] found that the methanolic extract from the fruits of *J. communis* exhibited moderate anti-tyrosinase activity. According to these authors, the most effective anti-tyrosinase compound in the extracts was the flavonoid hypolaetin 7-O-β-D-xylopyranoside. Furthermore, Jeong et al. [32] demonstrated that this flavonoid inhibited melanogenesis in a dose-dependent manner in a zebrafish model under in vivo conditions [32]. However, studies on the anti-tyrosinase activity of individual EOs have been carried out only to a limited extent, primarily focusing on in vitro research [14,33,34]. In addition, there are no available reports regarding the testing of the anti-tyrosinase activity of essential oil mixtures.

It has been revealed that extracts derived from the tissues of certain plant species (e.g., *Aerva lanata*, *Bruguiera gymnorhiza*, *Dodonaea viscosa*, *Lonicera japonica*, and *Schisandra chinensis*) exhibited anti-tyrosinase activity [35]. In addition, the study by Salihu et al. [34] demonstrated moderate anti-tyrosinase activity of the EO extracted from *Knema intermedia* Warb. Molecular docking analyses of this EO revealed that δ-selinene exhibited the lowest docking energy. It was also shown that this compound interacts with His85, His263, and His244 in the active site of tyrosinase [34]. Based on available studies, the EO isolated from *Rosa* × *damascena* Herrm. has been shown to reduce both intracellular tyrosinase activity and melanin content in the B16F10 murine melanoma cell line [14]. Detailed analyses revealed that this EO induced several structural changes in the tyrosinase enzyme under in vitro conditions, including a reduction in the content of α-helices, β-turns, and random coils, along with an increase in β-sheet content. Additionally, investigations conducted by Ha et al. [33] demonstrated that the EO extracted from the seeds of *Camellia japonica* exhibited anti-tyrosinase activity and suppressed melanogenesis in B16F10 melanoma cells. Furthermore, Murray et al. [36] found that the essential oil (EO) isolated from the leaves of *Polygonum odoratum* exhibited anti-tyrosinase activity. Single-compound assays revealed that three major constituents of this EO (i.e., dodecanal, decanal, and anisaldehyde) possessed significant anti-tyrosinase potential. According to the authors, interactions between the head and tail fragments of the alkanals may contribute to significant inhibition of tyrosinase activity. Another study conducted by Chang et al. [37] demonstrated that the EO derived from *Cinnamomum cassia* Presl also exerted an inhibitory effect on tyrosinase activity. Moreover, two major components of this EO, *cis*-2-methoxycinnamic acid and cinnamaldehyde (approx. 43% and 42%, respectively), were identified. However, in vitro studies did not confirm the anti-tyrosinase activity of *cis*-2-methoxycinnamic acid, while cinnamaldehyde was shown to exert an inhibitory effect [37]. Terpenoids, a diverse class of naturally occurring organic compounds predominantly found in EOs, have emerged as important bioactive agents with the ability to inhibit tyrosinase activity. Essential oils rich in terpenoids have demonstrated significant inhibitory effects on tyrosinase through various mechanisms [38,39,40]. For instance, monoterpenoids such as citronellol, geraniol, and limonene, commonly present in floral and citrus essential oils, exhibit reversible mixed-type inhibition, interacting with both the free enzyme and the enzyme–substrate complex [41,42]. This dual mode of inhibition not only reduces enzyme activity but may also contribute to decreased melanin synthesis with prolonged use [40,43]. Additionally, the sesquiterpenoid β-caryophyllene has been identified as interacting with the active site of tyrosinase, acting as a competitive inhibitor [44]. The molecular structures of terpenoids, characterized by hydrophobicity and the presence of functional groups such as aldehydes and alcohols, facilitate their binding affinity to the tyrosinase active site or allosteric sites, thereby modulating enzymatic activity. Beyond direct enzyme inhibition, terpenoid-rich EOs exhibit antioxidant properties, which may indirectly contribute to skin lightening by reducing oxidative stress and preventing the accumulation of reactive quinones that propagate melanogenesis [40,41,42].

The observed slight increase in tyrosinase activity over time at a low concentration (5 μg/mL) of the essential oil from *A. calamus* rhizomes (EOA) may be attributed to several time-dependent biochemical interactions. First, certain components of EOA may act as delayed effectors, requiring time to dissolve, diffuse, or interact with the enzyme to exert their modulatory effects on tyrosinase [37]. Second, oxidative transformation of volatile constituents within EOA during incubation may lead to the formation of secondary products that possess reduced inhibitory or even stimulatory effects on the enzyme [45]. Third, some constituents might contribute to the stabilization of the tyrosinase enzyme, preventing its degradation and thus allowing a greater proportion of active enzyme to persist during longer incubations [46]. Fourth, EOA components could improve the solubility or dispersion of the substrate in the assay medium over time, facilitating better substrate accessibility and consequently enhancing enzymatic activity [47]. Furthermore, at low EOA concentrations, a potential synergism between oil constituents and the tyrosinase–substrate complex may enhance enzyme activity. On the other hand, at higher oil concentrations, hydrophobic microstructures such as emulsions or micelles may form and hinder substrate diffusion; in contrast, at lower concentrations, these structures may disassemble or diffuse, allowing improved enzymatic efficiency.

### 2.3. Antioxidant Capacity of the Examined Essential Oils

**Figure 3:**
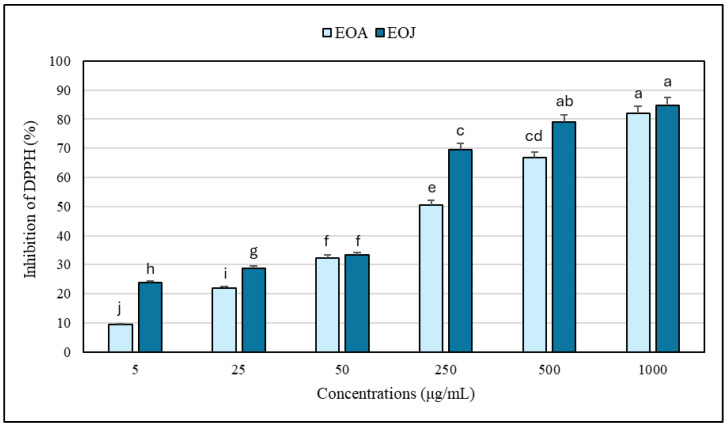
Antioxidant activity of the studied essential oils against DPPH free radicals. EOA and EOJ—essential oils extracted from *Acorus calamus* L. rhizomes and *Juniperus communis* L. cone-berries, respectively. Distinct letters above the error bars indicate significant differences in the means (±SD) of antioxidant activities between the samples, as determined by Tukey’s test (*p* < 0.05).

**Figure 4:**
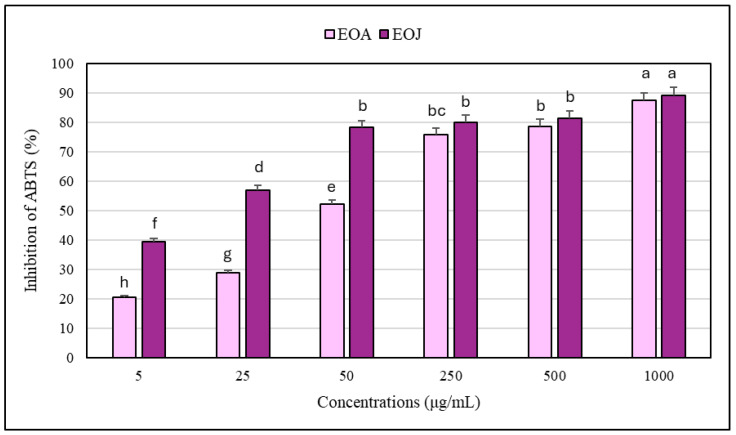
Antioxidant activity of the investigated essential oils against ABTS free radicals. EOA and EOJ—essential oils extracted from *Acorus calamus* L. rhizomes and *Juniperus communis* L. cone-berries, respectively. Distinct letters above the error bars indicate significant differences in the means (±SD) of antioxidant activities between the samples, as determined by Tukey’s test (*p* < 0.05).

The results regarding the antioxidant potential of the two tested EOs and their mixtures determined by the DPPH and ABTS free radical scavenging methods are shown in Figure 3 and Figure 4. Increasing concentrations of ethanolic solutions (i.e., 5, 25, 50, 250, 500, 1000 μg/mL) of the two tested EOs showed progressively higher degrees of antioxidant activity. The EOA solutions caused 9–82% and 21–87% inhibition of DPPH^•^ and ABTS^•+^, respectively, whereas the EOJ samples exhibited 24–85% and 39–89% inhibition of the tested free radicals, respectively (Figure 3 and Figure 4). In addition, all the examined concentrations of essential oil isolated from *J. communis* cone-berries exhibited a stronger antioxidative potential than the other analyzed EO extracted from *A. calamus* rhizomes (i.e., 1–19% and 2–28% higher inhibition of DPPH^•^ and ABTS^•+^, respectively) (Figure 3 and Figure 4). Moreover, the IC_50_ values for the EOA were determined to be 216.5 ± 1.4 μg/mL and 45.0 ± 0.3 μg/mL against DPPH^•^ and ABTS^•+^ radicals, respectively. In turn, the IC_50_ values determined for the other tested essential oil (i.e., EOJ) were 85.4 ± 0.8 μg/mL and 14.2 ± 0.1 μg/mL against DPPH^•^ and ABTS^•+^ radicals, respectively.

**Figure 5:**
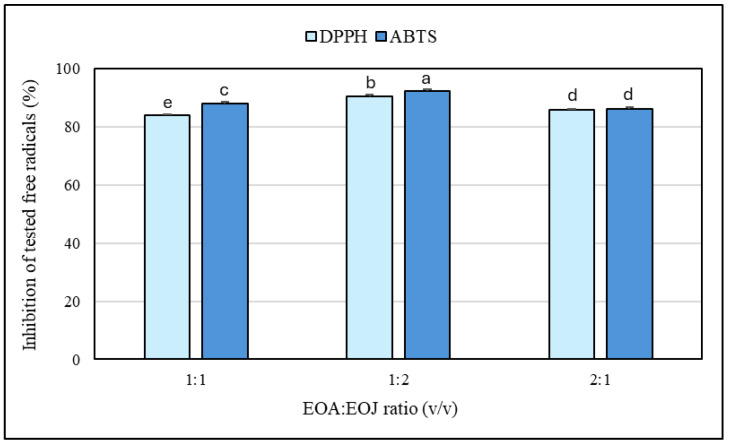
Antioxidant activity of the mixtures of the two examined essential oils. The proportions 1:1, 1:2, and 2:1 refer to the volume ratios of EOA to EOJ (*v*/*v*) in the tested mixtures. EOA and EOJ—essential oils extracted from *Acorus calamus* L. rhizomes and *Juniperus communis* L. cone-berries, respectively. Distinct letters above the error bars indicate significant differences in the means (±SD) of antioxidant activities between the samples, as determined by Tukey’s test (*p* < 0.05).

Furthermore, evaluation of both essential oil mixtures at the highest tested concentration (1 mg/mL) indicated that the ratio of 1:2 (*v*/*v*) EOA:EOJ exhibited the greatest antioxidant activity (90–92% inhibition of DPPH^•^ or ABTS^•+^), whereas the 1:1 and 2:1 ratios were slightly less effective (83–88% inhibition) (Figure 5). In addition, the antioxidant potential of the studied EOs was found to be higher against ABTS^•+^ compared to DPPH^•^ (i.e., 6–25% and 2–45% higher inhibition of the radicals in the case of EOA and EOJ, respectively, as well as 0.3–4% greater inhibition for the tested mixtures of the two EOs) (Figure 3 and Figure 4). As shown in Table 4, all tested mixtures (1:1, 1:2, and 2:1 volume ratio of EOA to EOJ) resulted in CI values below 1, indicating synergistic interactions in antioxidant activity against both DPPH and ABTS radicals. For the DPPH^•^ assay, CI values ranged from 0.847 (1:2 ratio) to 0.944 (2:1 ratio), suggesting slight synergism to near-additive effects. In the ABTS^•+^ assay, CI values were slightly lower, ranging from 0.718 (1:2 ratio) to 0.850 (2:1 ratio), indicating moderate synergism, particularly for the 1:2 mixture. Among the tested combinations, the 1:2 ratio (with a higher proportion of EOJ) consistently demonstrated the most pronounced synergistic interaction in both assays. These findings suggest that combining EOA and EOJ enhances antioxidant activity more effectively than individual essential oils, with the magnitude of synergy depending on both the mixture ratio and the radical system used.

DPPH^•^ and ABTS^•+^ radicals are widely used to assess the antioxidant potential of essential oils (EOs) derived from a variety of plant sources [7,24,48]. Evaluating free radical scavenging activity is particularly important when examining essential oils intended for topical application to the skin (e.g., as bath additives, creams, gels, lotions, hydrolats, etc.). Our results demonstrate the high antioxidant potential of both individual and mixed EOs extracted from *A. calamus* and *J. communis*. Several reports have shown that the content of bioactive compounds in EOs from various plant species is strongly influenced by a wide range of environmental factors [5,6,7]; therefore, a detailed comparison of antioxidant potential between EOs, even those derived from the same plant species, is difficult and carries notable limitations. Studies conducted by Loying et al. [24] revealed that the EO isolated from *A. calamus* rhizomes in Northeast India exhibited high antioxidant activity against DPPH^•^, whereas Khanal et al. [48] found that the EOA isolated from plants grown in Nepal displayed relatively lower activity against the tested radicals. According to Höferl et al. [8], the EO extracted from *J. communis* plants grown in Bulgaria (α-pinene-rich chemotype) showed lower activity against DPPH^•^ (IC_50_ = 34.80 mg/mL) and significantly higher antioxidant potential against ABTS^•+^ (IC_50_ = 10.96 µg/mL). However, the inhibition of lipid peroxidation by this EO was considerably less effective [8]. It has been found that α-pinene did not exhibit scavenging activity against DPPH^•^, β-pinene and limonene displayed only very low antioxidant activity (below 1%), whereas α-thujene showed approximately 5% activity [49].

In addition, Assaggaf et al. [50] revealed that a mixture of three EOs isolated from the aerial parts of *A. calamus* (asarone-rich chemotype), *Cymbopogon flexuosus* L., and the seeds of *Carum carvi* L., combined in different proportions, led to an increase in antioxidant potential compared to the individual EOs. We demonstrated that a mixture of the tested essential oils in a 1:2 (EOA:EOJ) ratio exhibited slightly stronger antioxidant activity compared to the two other mixture ratios and the individual EOs. This may be due to several factors, including additive or synergistic interactions between the chemical constituents of both EOs. For example, some components may enhance the activity of others, thereby increasing the scavenging capacity against DPPH^•^ and/or ABTS^•+^. It should also be considered that the chemical components of EOs may act through diverse mechanisms, resulting in enhanced antioxidant potential. A mixture of EOs offers a broader range of molecules with varying polarity, structure, size, and accessibility to free radicals. Another contributing factor could be the stability of certain chemical compounds, some of which may be more stable when the EOs are combined in optimal proportions than when present in individual oils. On the other hand, the higher antioxidant potential of the individual and combined essential oils observed using the ABTS method, compared to the DPPH assay, may be attributed to, among other factors, different reaction mechanisms of EO constituents with DPPH^•^ and ABTS^•+^ radicals. These differences are related to the chemical properties of the EO components and the reaction environment. Furthermore, the DPPH method may be less sensitive to compounds with limited solubility in organic solvents (e.g., ethanol or methanol), which may affect the accuracy of the antioxidant potential determination.

## 3. Materials and Methods

### 3.1. Plant Materials and Extraction of the Examined Essential Oils

The essential oils (EOs) were derived from the rhizomes of *Acorus calamus* L. and the cone-berries of *Juniperus communis* L. The dried plant materials were obtained from a local herbal store (NANGA, Blękwit, Poland), and the supplier provided quality certificates for the tested specimens, confirming compliance with acceptable standards for moisture and ash content, heavy metals, absence of ochratoxin A, appropriate organoleptic properties, as well as permissible levels of aerobic bacteria and fungi.

The plant materials under study were ground into a fine powder and sieved to obtain particles of 20–40 mesh size. Subsequently, 40 g portions were weighed and subjected to essential oil extraction for 3 h using the hydrodistillation method with a Clevenger-type apparatus. After the extraction was completed, the EOs were collected, dried over anhydrous sodium sulfate, filtered, and stored at 4 °C in tightly sealed vials until further analyses were performed [51].

In order to evaluate the antioxidant and anti-tyrosinase potential of the tested essential oils, the following dilutions of EO samples in ethanol were prepared: 5, 25, 50, 250, 500, and 1000 µg/mL (*w*/*v*). Additionally, three mixtures of the two tested EOs were prepared at a concentration of 1 mg/mL, in the following volume ratios of EOA to EOJ (*v*/*v*): 1:1, 1:2, and 2:1.

### 3.2. Analysis of Chemical Compounds in the Tested Essential Oils Using Gas Chromatography–Mass Spectrometry (GC-MS)

The composition of the two EOs studied was analyzed using a TRACE GC-ULTRA gas chromatograph coupled with a Polaris Q mass spectrometer (Thermo Fisher Scientific, Waltham, MA, USA). The analyses were performed using an Equity-5 capillary GC column (Supelco) with a length of 30 m, an internal diameter of 0.25 mm, and a film thickness of 0.25 μm. The thermal profile ranged from 40 °C to 280 °C, with a temperature increase of 5 °C/min. The injection port and transfer line temperatures were set to 280 °C. The flow rate of helium (the carrier gas) was set to 1 mL/min. Samples of the studied EOs (1 μL each) were injected. Electron impact ionization (70 eV) was performed, with a full-scan acquisition in the *m*/*z* range of 35–400. To identify the separated chemical constituents of the EOs, the obtained retention indices (RIs) and mass spectra were compared with those reported in the literature and in the NIST Mass Spectral Library (National Institute of Standards and Technology, Gaithersburg, MD, USA) [52,53].

### 3.3. Determination of Anti-Tyrosinase Potential

Evaluation of the inhibitory potential of the essential oils (EOs) against tyrosinase activity was measured using the Tyrosinase Inhibitor Screening Assay Kit (catalogue no. ab204715, Abcam, Cambridge, UK), following the manufacturer’s protocol. Each EO sample was prepared at the specified concentration by diluting it in a premix consisting of 2 µL of 96% ethanol, 1 µL of DMSO (dimethyl sulfoxide), and Tyrosinase Assay Buffer to a total volume of 20 µL. The reaction mixture was then assembled by combining the 20 µL EO sample with 50 µL of Tyrosinase Enzyme Solution and 30 µL of Tyrosine/Tyrosinase Substrate Solution, resulting in a total assay volume of 100 µL. This preparation yielded final solvent concentrations of approximately 1.92% (*v*/*v*) ethanol and 1% (*v*/*v*) DMSO. In addition, an Enzyme Control (EC), containing no EO sample, was included. Kojic acid, dissolved in double-distilled water (ddH_2_O) at a concentration of 140 µg/mL, was used as the Inhibitor Control (IC). The Solvent Control (SC) consisted of 2 µL of 96% ethanol, 1 µL of DMSO, and 17 µL of Tyrosinase Assay Buffer (volume of 20 µL). After addition of the SC to the reaction mixture (total volume of 100 µL), the final solvent concentrations were approximately 1.92% (*v*/*v*) ethanol and 1% (*v*/*v*) DMSO. All the reaction mixtures were incubated at 25 °C for 60 min, and then the absorbance at 510 nm was measured, using a microplate UV-Vis spectrophotometer (Epoch, BioTek, Winooski, VT, USA). The degree of enzyme inhibition was calculated using the following formula:

% Relative Inhibition = [(Slope of EC − Slope of S)/Slope of EC] × 100,

where EC—enzyme control, and S—sample.

### 3.4. Measurement of Antioxidant Capacity

#### 3.4.1. DPPH^•^ Scavenging Assay

The antioxidant potential of the tested essential oils against DPPH^•^ (1,1-diphenyl-2-picrylhydrazyl) was determined using the modified method of Brand-Williams et al. [54]. A stock solution was prepared by dissolving 9.8 mg of DPPH^•^ in 50 mL of ethanol (96%; *v*/*v*). Then, 4 mL of DPPH^•^ stock solution was diluted with 8 mL of 96% ethanol and vortexed vigorously for 1 min. The reaction mixture contained 350 μL of DPPH^•^ working solution and 50 μL of the tested sample of EO. The mixtures were vortexed for 15 s and then incubated in the dark at room temperature for 30 min. Using a UV-Vis microplate spectrophotometer (Epoch, BioTek, Winooski, VT, USA), the absorbance (λ = 517 nm) of the DPPH^•^ solution (A_0_) and the tested samples of essential oils (A_S_) was measured. Immediately before analysis, the absorbance of the DPPH^•^ solution was adjusted to 1.0 by adding 96% ethanol. The antioxidant potential of the tested ethanol dilutions of the EOs was calculated using the following formula:

DPPH^•^ inhibition (%) = [(A_0_ − A_S_)/A_0_] × 100

where A_0_—absorbance of DPPH^•^ solution, and A_S_—absorbance of EO sample tested.

Furthermore, the IC_50_ value was assessed, representing the concentration of the examined EO (μg/mL) required to cause 50% inhibition of DPPH^•^.

#### 3.4.2. ABTS^•+^ Scavenging Assay

The antioxidant capacity of the studied EOs against ABTS^•+^ (2,2′-azinobis(3-ethylbenzothiazoline-6-sulfonic acid) was evaluated using the modified method of Re et al. [55]. The generation of ABTS^•+^ was conducted by mixing an aqueous solution of ABTS (7 mM) and potassium persulfate (2.45 mM) in a 1:0.5 ratio. Then, this mixture was incubated in the dark at room temperature for 16 h. Before performing analyses, the stock solution of ABTS^•+^ was diluted with 96% ethanol to achieve an absorbance of 0.7. The reaction mixture consisted of 350 μL of ABTS^•+^ working solution and 50 μL of the EO sample. The mixtures were vortexed for 15 s and then incubated in the dark at room temperature for 30 min. Using a UV-Vis microplate spectrophotometer (Epoch, BioTek, Winooski, VT, USA), the absorbance (λ = 734 nm) of the ABTS^•+^ solution (A_0_) and the tested samples of essential oils (A_S_) was measured. The antioxidant potential of the tested ethanolic dilutions of the EOs was determined from the following formula:

ABTS^•+^ inhibition (%) = [(A_0_ − A_S_)/A_0_] × 100

where A_0_—absorbance of ABTS^•+^ solution, and A_S_—absorbance of EO sample tested.

In the next step, the IC_50_ value was determined, representing the concentration of the tested EO (μg/mL) causing 50% inhibition of ABTS^•+^.

### 3.5. Determination of Combination Index of Essential Oil Mixtures

To evaluate the interaction effects between the essential oils from *A. calamus* rhizomes and *J. communis* cone-berries (EOA and EOJ, respectively), we applied the median-effect method developed by Chou and Talalay [30,56]. EO combinations were tested at fixed ratios (1:1, 1:2, and 2:1; *v*/*v*), and dose–response interactions were established for each mixture, as well as for individual EOs. The Combination Index (CI) was calculated to quantify the nature of the interactions, where CI < 1 denotes synergism, CI = 1 indicates an additive effect, and CI > 1 reflects antagonism [30]. The CI values for the tested mixtures of EOA and EOJ were determined using the following equation:

CI = (D1/D_x1_) + (D2/D_x2_)

where D1 and D2 represent the concentrations of EOA and EOJ, respectively, in a combined mixture that contributes to achieving a given inhibitory effect; and D_x1_ and D_x2_ represent the concentrations of EOA and EOJ when tested individually that contribute to achieving a given inhibitory effect.

### 3.6. Statistical Analyses

Data are presented as the mean (±SD; standard deviation). Three independent series of experiments and two technical replicates were carried out. The significance of the differences in the levels of the examined parameters (i.e., anti-tyrosinase potential, antioxidant capacity) of two EOs and their mixtures was calculated using an analysis of variance (ANOVA) with subsequent post hoc Tukey’s test. *p*-values less than 0.05 were considered significant. All statistical analyses were performed using Statistica ver. 13.3 software (Statsoft, Kraków, Poland).

## 4. Conclusions

The essential oil extracted from *J. communis* cone-berries (EOJ) exhibited a significantly higher diversity of chemical components compared to the other examined essential oil isolated from *A. calamus* rhizomes (EOA). The GC-MS analyses identified an acorenone-rich chemotype of the EOA and an *α*-pinene-rich chemotype of the EOJ. We have provided evidence that both EOs display high antioxidant capacity and effectively inhibit tyrosinase activity. Therefore, additional research is recommended to evaluate these activities in human cell lines and in vivo models, with a focus on determining the optimal concentrations of the tested EOs. However, the increase in anti-tyrosinase activity observed at a low concentration of EOA warrants particular attention in subsequent studies. The next stages of investigation should include both the cytotoxicity and genotoxicity screening of the tested EOs, both as single formulations and in combination.

## Figures and Tables

**Table 1:** GC-MS identification and quantification of chemical compounds in the essential oil from *Acorus calamus* (L.) rhizomes.

Chemical Constituents	RT	RI	Peak Area (%)
*α*-Pinene	11.00	931	0.4
Camphene	11.56	946	2.1
Sabinene	12.55	971	0.1
*β*-Pinene	12.64	974	0.1
α-Phellandrene	13.75	1003	<0.1
*p*-Cymene	14.53	1023	0.1
Limonene	14.69	1027	0.1
1,8-Cineole	14.77	1029	0.1
Linalool	17.46	1099	0.3
Camphor	19.09	1143	3.5
*α*-Copaene	27.27	1377	0.2
*β*-Elemene	27.80	1393	0.2
*β*-Cedrene	28.53	1416	2.2
Aristolene	28.68	1421	1.6
*β*-Copaene	28.74	1423	1.1
*cis*-Thujopsene	28.98	1431	0.2
*β*-Gurjunene	29.11	1435	4.0
(*E*)-*α*-Bergamotene	29.18	1437	0.8
Prezizaene	29.57	1450	0.9
Zizaene	29.73	1455	2.6
*α*-Acoradiene	30.19	1469	0.2
*α*-Neocallitropsene	30.38	1475	0.5
Ledene	30.84	1490	1.0
6-Epishyobunone	30.99	1495	2.0
*α*-Selinene	31.08	1498	4.0
Shyobunone	31.63	1516	7.5
*δ*-Cadinene	31.91	1526	1.0
Isoshyobunone	32.10	1532	5.7
*α*-Calacorene	32.51	1546	2.5
*β*-Calacorene	33.13	1567	0.6
Spathulenol	33.54	1581	1.3
Caryophyllene oxide	33.71	1586	0.8
Cedrol	34.27	1606	0.8
Preisocalamendiol	34.38	1610	12.0
1,2-Humulenepoxide	34.48	1613	1.6
*β*-Asarone	34.81	1625	4.2
Dehydroisocalamendiol	35.08	1634	2.7
*Epi*-*α*-muurolol	35.41	1646	0.3
1-Isopropyl-4,8-dimethylspiro[4.5]decan-7-one	35.50	1649	2.4
*α*-Cadinol	35.76	1658	0.9
10*β*-Hydroxy-*cis*-calamenene	35.87	1662	0.9
10*α*-Hydroxy-*cis*-calamenene	36.12	1671	0.4
*α*-Asarone	36.43	1682	0.6
Acorenone	36.74	1693	18.1
Acora-7(11),9-dien-2-one	37.69	1728	0.2
Isocalamendiol	38.24	1749	3.0
Acorone isomer 1	39.92	1812	0.5
Acorone isomer 2	40.45	1832	0.3
**Total**	-	-	**96.6**

GC-MS—gas chromatography–mass spectrometry; RT—retention time (min.); RI—retention index.

**Table 2:** GC-MS identification and quantification of chemical compounds in the essential oil from *Juniperus communis* (L.) cone-berries.

Chemical Constituents	RT	RI	Peak Area (%)
Tricyclene	10.55	919	0.1
*α*-Thujene	10.79	926	0.5
*α*-Pinene	11.00	931	22.1
Camphene	11.56	946	0.3
2,4(10)-Thujadiene	11.79	952	<0.1
Sabinene	12.55	971	2.3
*β*-Pinene	12.64	974	1.8
*β*-Myrcene	13.30	991	3.2
Δ3-Carene	13.98	1009	<0.1
*p*-Cymene	14.53	1023	3.6
Limonene	14.69	1027	2.3
1,8-Cineole	14.77	1029	0.1
(*Z*)-Sabinene hydrate	16.19	1066	0.1
*α*-Pinene oxide	17.35	1096	1.1
Perillene	17.50	1100	0.2
*α*-Fenchol	17.94	1112	<0.1
*β*-Thujone	18.04	1115	0.2
Dehydrosabinaketone	18.15	1118	0.1
(*Z*)-*p*-Menth-2-en-1-ol	18.24	1120	0.2
*α*-Campholenal	18.42	1125	0.4
*cis*-Limonene-oxide	18.69	1132	<0.1
*trans*-Pinocarveol	18.89	1138	0.9
*cis*-Verbenol	19.13	1144	1.3
(*E*)-*p*-Menth-2-en-1-ol	19.24	1147	0.1
Pinocarvone	19.77	1162	0.3
Borneol	19.90	1165	0.4
Terpinen-4-ol	20.33	1177	3.3
*p*-Cymen-8-ol	20.62	1185	1.0
*α*-Terpineol	20.82	1190	0.7
Myrtenal	21.01	1195	0.5
Methyl chavicol	21.10	1198	0.5
Verbenone	21.48	1208	2.0
*trans*-Carveol	21.84	1219	0.3
Cuminyl aldehyde	22.57	1239	0.1
Carvone	22.72	1244	0.2
*p*-Anisaldehyde	23.07	1254	1.6
*p*-Menth-2-en-1,4-diol	23.60	1269	0.1
*trans*-Anethol	24.17	1285	0.6
Bornyl acetate	24.21	1286	0.7
2-Undecanone	24.45	1293	0.2
*α*-Cubebene	26.38	1351	1.1
Eugenol	26.62	1358	0.1
*α*-Ylangene	27.12	1373	0.1
*α*-Copaene	27.27	1377	1.5
*β*-Elemene	27.80	1393	2.1
Bicyclo-4(15)-oppositene	28.24	1407	0.1
(*E*)-*β*-caryophyllene	28.69	1421	0.4
*β*-Copaene	28.99	1431	0.1
*γ*-Elemene	29.12	1435	0.1
(*E*)-*α*-Bergamotene	29.18	1437	0.1
Guaia-6,9-diene	29.39	1444	0.1
*α*-Caryophyllene	29.77	1456	0.7
*γ*-Muurolene	30.48	1479	1.5
*β*-Selinene	30.81	1489	1.3
*epi*-Cubebol	31.06	1497	0.7
*γ*-Cadinene	31.64	1517	1.9
*trans*-Calamenene	31.90	1525	0.5
Selina-3,7(11)-diene	32.29	1539	0.1
Nootkatene	32.34	1540	0.2
α-Calacorene	32.47	1545	0.2
Elemol	32.68	1552	0.3
*β*-Calacorene	33.06	1565	0.2
Mintoxide	33.22	1570	0.2
Spathulenol	33.54	1581	3.1
Caryophyllene oxide	33,71	1586	3.7
4(14)-Salvialene-1-one	34.02	1597	0.4
*trans*-Caryophyllene oxide	34.18	1602	0.2
1,2-Humulenepoxide	34.48	1613	3.4
1-*epi*-Cubenol	35.00	1631	1.0
*epi*-*α*-Cadinol	35.39	1645	1.8
*α*-Muurolol	35.51	1649	0.5
*β*-Eudesmol	35.64	1654	0.2
*α*-Cadinol	35.76	1658	2.3
10*β*-Hydroxy-*cis*-calamenene	35.87	1662	0.4
ar-Turmerone	35.99	1666	0.2
10*α*-Hydroxy-*cis*-calamenene	36.12	1671	0.7
Cadalene	36.32	1678	0.4
Oplopanone	38.04	1741	0.3
Benzyl benzoate	38.70	1766	0.1
Manoyl oxide	44.55	1997	0.1
Abietatriene	46.04	2060	0.4
**Total**	-	-	**85.7**

GC-MS—gas chromatography–mass spectrometry; RT—retention time (min.); RI—retention index.

**Table 3:** Combination Index (CI) values and interaction effects of the essential oil mixtures (EOA and EOJ) on tyrosinase activity.

Mixture Ratio (EOA:EOJ)	CI	Effect of Interaction
1:1	0.805	Synergism
1:2	0.920	Slight Synergism/Near-Additive
2:1	0.904	Slight Synergism/Near-Additive

EOA and EOJ—essential oils extracted from *Acorus calamus* L. rhizomes and *Juniperus communis* L. cone-berries, respectively. The proportions 1:1, 1:2, and 2:1 refer to the volume ratios of EOA to EOJ (*v*/*v*) in the tested mixtures. CI—Combination Index, calculated according to the median-effect principle and the Chou–Talalay method [30]. CI values were interpreted as follows: CI < 1 denotes synergistic interaction, CI = 1 indicates an additive effect, and CI > 1 reflects antagonism.

**Table 4:** Combination Index (CI) values and interaction effects of the essential oil mixtures (EOA and EOJ) on antioxidant activity against DPPH and ABTS radicals.

Mixture Ratio (EOA:EOJ)	DPPH^•^		ABTS^•+^	
	CI	Effect of Interaction	CI	Effect of Interaction
1:1	0.910	Slight Synergism/Near-Additive	0.815	Synergism
1:2	0.847	Synergism	0.718	Synergism
2:1	0.944	Slight Synergism/Near-Additive	0.850	Synergism

EOA and EOJ—essential oils extracted from *Acorus calamus* L. rhizomes and *Juniperus communis* L. cone-berries, respectively. The proportions 1:1, 1:2, and 2:1 refer to the volume ratios of EOA to EOJ (*v*/*v*) in the tested mixtures. CI—Combination Index, calculated according to the median-effect principle and the Chou–Talalay method [30]. CI values were interpreted as follows: CI < 1 denotes synergistic interaction, CI = 1 indicates an additive effect, and CI > 1 reflects antagonism.

## Data Availability

The data presented in the study are available in the article.

## Supplementary Materials

The following supporting information can be downloaded at: https://www.mdpi.com/article/10.3390/molecules30112417/s1, Figure S1: GC-MS chromatogram of the essential oil extracted from *Acorus calamus* (L.) rhizomes; Figure S2: GC-MS chromatogram of the essential oil extracted from *Juniperus communis* (L.) cone-berries.
