# Supplementary material for: Chemical Composition, Anti-Tyrosinase and Antioxidant Potential of Essential Oils from Acorus calamus (L.) and Juniperus communis (L.)
Source: Molecules. 2025 May 31;30(11):2417. doi: 10.3390/molecules30112417 (PMC12156826; doi:10.3390/molecules30112417)
Supplement: Supplementary file 1 [file molecules-30-02417-s001.zip › Figure S2.pdf]

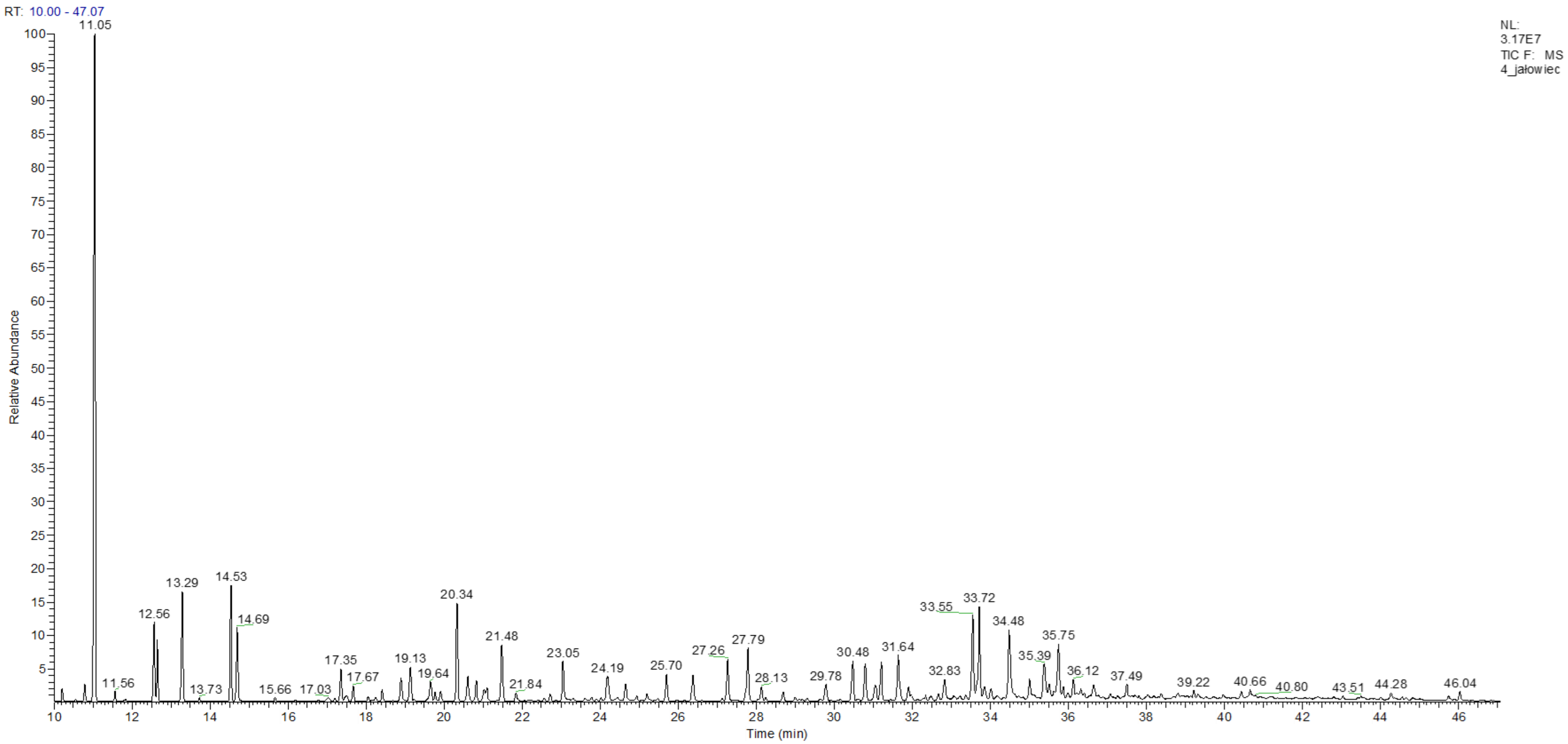

Figure S2. GC-MS chromatogram of the essential oil extracted from *Juniperus communis* (L.) cone-berries.
